# Hypoxic Stress Induces Complement-Mediated Lysis of Mesenchymal Stem Cells by Downregulating Factor H and CD59

**DOI:** 10.1007/s13770-024-00678-6

**Published:** 2024-11-01

**Authors:** Ramada R. Khaswaneh, Ejlal Abu-El-Rub, Ayman Alzu’bi, Fatimah A. Almahasneh, Rawan. A. Almazari, Heba F. AI-jariri, Raed M. Al-Zoubi

**Affiliations:** 1 Department of Basic Medical Sciences, Faculty of Medicine, Yarmouk University, Irbid, 211-63 Jordan; 2 Surgical Research Section, Department of Surgery, Hamad Medical Corporation, Doha, Qatar; 3 Department of Biomedical Sciences, QU-Health, College of Health Sciences, Qatar University, 2713 Doha, Qatar; 4 Department of Chemistry, Jordan University of Science and Technology, P.O.Box 3030, Irbid, 22110 Jordan

**Keywords:** MSCs, Hypoxia, Survival, Factor H, CD59, MAC complex, Apoptosis

## Abstract

**Background:**

Factor H and membrane inhibitor of reactive lysis (CD59) are key regulators of complement activation. Mesenchymal stem cells (MSCs) secrete Factor H and express CD59 to protect themselves from complement-mediated damage. Severe hypoxia found to decrease the survival chances of MSCs after transplantation; however, little is known about the impact of severe hypoxia on modulating the complement system activity and its effect on MSCs survival. Our study seeks to explore the effect of severe hypoxia on modulating the complement cascade in MSCs.

**Methods:**

Human adipose tissue-derived MSCs (hAD-MSCs) were cultured under severe hypoxia using 400 μM Cobalt Chloride (CoCl2) for 48 h. The protein expressions of survival marker; Phosphoinositide 3-kinases (PI3K), and pro-apoptotic marker; Caspase-3 were assessed using western blotting. The level of complement system related factors; Factor H, CD59, C3b, iC3b, C5b, C9, and the complement membrane attack complex (MAC) were analyzed using Elisa assays, western blotting, and immunocytochemistry.

**Results:**

Our results showed for the first time that severe hypoxia can significantly impair Factor H secretion and CD59 expression in MSCs. This has been associated with upregulation of MAC complex and increased level of cell lysis and apoptosis marked by downregulation of PI3K and upregulation of Annexin v and Caspase-3.

**Conclusion:**

The loss of Factor H and CD59 in hypoxic MSCs can initiate their lysis and apoptosis mediated by activating MAC complex. Preserving the level of Factor H and CD59 in MSCs has significant clinical implication to increase their retention rate in hypoxic conditions and prolong their survival.

**Supplementary Information:**

The online version contains supplementary material available at 10.1007/s13770-024-00678-6.

## Introduction

Mesenchymal stem cells (MSCs) are considered the ideal candidates among all other types of stem cells for regenerating injured tissues and restore the functionality of damaged organs [1]. These stem cells possess remarkable ability to abide many hostile microenvironments existing at the site of transplantation, including inflammation, hypoxia, and oxidative stress. This can be done by activating specific signaling pathways that can increase MSCs’ endurance and survival in stressful microenvironments. Complement Factor H and MAC-inhibitory protein (CD59) are among the factors released and expressed by MSCs to facilitate their survival and integration at the host site. Factor H and CD59 are potent regulators of the complement system and participate in suppressing its activation [2].

The complement system is a fundamental component of the innate immune system that acts rapidly in defending the body against pathogens and foreign molecules [3]. Apart from its defensive functions, the complement system plays an uppermost role in preserving tissue homeostasis by purging apoptotic cells and cellular debris which is crucial to prevent further tissue damage and infection spread [4, 5].

Activating the complement system can trigger cellular lysis mediated by the assembly of membrane-penetrating pores known as the membrane attack complex (MAC). Further, the activation of complement system is associated with opsonization/phagosytosis and the release of anaphylatoxins that intensify the local inflammatory mechanism [6].

There are three known pathways that belong to complement system: classical, lectin, and alternative pathways [7]. All three pathways converge and activate C3 convertase which cleaves the C3 protein into C3a and C3b. C3b also binds the C3 convertase to form C5 convertase which cleaves C5 factor into C5a and C5b inflammatory peptides [8]. The C5b fragment interacts with multiple factors; C6, C7, C8 and C9, forming C5b-9 or MAC complex. MAC is the final product of all three complement pathways that induces cellular lysis and triggers apoptosis [7, 9].

Upregulating the levels of Factor H and CD59 in MSCs is considered a protective mechanism to exert an anti-inflammatory response and inhibit the activation of complement system. Factor H and CD59 protect MSCs from complement-mediated damage, thus enhance their survival and adaptive abilities. Through inhibiting the formation and stabilization of C3 convertase, Factor H and CD59 prevent the activation of the subsequent downstream steps in the complement cascade and cease the formation of pro-apoptotic MAC complex [2, 10].

Recent studies suggested that MSCs may be exterminated when placed in severe hypoxic microenvironment [12, 13]. Severe hypoxia can alter the functional properties of MSCs, particularly diminishing their abilities to effectively suppress immune response causing poor survival of MSCs in such stressful condition [14]. Besides, severe hypoxia can increase the apoptosis of MSCs at the site of transplantation and decrease the availability of MSCs to perform regenerative functions [14]. The impact of severe hypoxia on triggering complement –mediated damage of MSCs remains largely unexplored. So the primary aim of this study is to investigate the effect of severe hypoxia on the levels of Factor H and CD59 as regulatory factors for complement system. Understanding the impact of severe hypoxia on modulating the complement system is crucial for optimizing MSCs-based therapy, especially in hypoxia-associated pathologies.

## Materials and methods

Cell line: Human Adipose tissue-derived MSCs (hAD-MSCs) was commercially purchased from Lonza company (Cat# PT5006, Lot# 21TL138912). MSCs were expanded using Dulbecco’s Modified Eagle’s Medium Low Glucose (DMEM-Low Glucose, Euroclone) which contained 5.6 mmol/L glucose and were supplemented with 10% fetal bovine serum (FBS, Gibco) [15], 0.1 mg/ml streptomycin, and 100 units/ml penicillin in standard cell culture incubator (5% CO2/95% air; 37 °C). Medium was changed every 72 h and cells were passaged when confluency was over 70%.

Hypoxia induction: Cobalt Chloride (CoCl2) was used as a chemical inducer of hypoxia [16]. CoCl2 solution was added in the MSCs culture media at a final concentration of 400 μm. MSCs were incubated in the culture media having CoCl2 for 48 h. MSCs grown in normoxic condition (5% CO2/95% air; 37 °C) were considered the control group.

### Reagents and chemicals

Antibodies used for Western blotting and Immunostaining: HIF-1α (Santa Cruz Biotechnology, US, Cat# sc-53546), TNF-α (Santa Cruz Biotechnology, Cat# sc-52746), PI3K (Santa Cruz Biotechnology, Cat # sc-1637), Caspase-3 (Santa Cruz Biotechnology, Cat # sc-56053), Factor H (MyBiosource Cat # MBS8565537), CD59 (MyBiosource Cat # MBS210610), C5b (MyBioSource, Cat # MBS530209), C9 (R & D system Cat # MAB8126), β-actin (Santa Cruz Biotechnology Cat # sc-47778 HRP), Phalloidin-iFluor 488 Reagent (Abcam, Cat # ab176753), Anti-mouse Alexa Fluor 647 (Invitrogen, USA), Anti-rabbit Alexa Fluor 647 (Invitrogen, USA), Mounting Medium With DAPI (Abeam, UK), Ultra High Sensitivity ECL Kit (GlpBio Cat# GK10008).

### Western blotting

The protein levels of PI3K, Caspase-3, Factor H, CD59, C5b, and C9 were measured by Western blotting. Briefly, hAD-MSCs were seeded in 10 cm dishes and cultured for 96 h until getting confluent, then they exposed to hypoxia as described above. The cells were scraped using cold phosphate buffered saline (PBS) and pelleted. The cells pellet was re-suspended in protein lysis buffer (RIPA with protease inhibitors). Total protein levels were measured using NanoDrop™ Lite Spectrophotometer, and 40 μg of protein was loaded onto SDS–PAGE. Following electrophoresis, proteins were transferred to PVDF membrane and were incubated with appropriate primary and secondary antibodies. The membranes were visualized using VILBER FUSION Gel Documentation System, and bands were quantified using ImageJ for densitometry and normalized to β-actin.

### Immunocytochemistry

hAD-MSCs cells were seeded onto sterile coverslips and allowed to grow till 60% confluency. After being exposed to hypoxic stress for 48 h, the plated cells were fixed with 4% paraformaldehyde and permeabilized using 0.2% Triton X in PBS at RT. The cells were then stained with respective primary and secondary fluorescent antibodies, Thereafter, the stained cells were preserved and counter-stained with Mounting Media having DAPI (4′,6-diamidino-2-phenylindole) for nuclei. The cells were imaged using Cytation 5 imaging system (BioTek, USA).

### ELISA assays

ELISA assays were performed to detect the expression and activity of C3b (Genochem world SL., lot # GW3605Hu), iC3b (My BioSource, Cat # MBS1603937), and MAC complex (C5b-C9) (GenoChem, Cat # GW3858Hu), following the manufacturer's instructions. Briefly, MSCs were seeded in 96 well plate (1X 105 cell/well) and allowed to attach overnight. The attached cells then exposed to hypoxic stress for 48 h, followed by performing the ELISA assays. The absorbance values which represent the level of each complement factor were detected using Cytation 5 (BioTek, USA).

### Statistical analysis

Data were reported as mean ± SD. Comparison of data between multiple groups was performed using one-way analysis of variance (ANOVA) followed by Tukey’s post-hoc multiple comparison test, and analysis between two groups was made using Student’s t-test (two-tailed). Statistical significance is determined as p < 0.05. Each figure represents one of at least three independent quantifiable experiments.

## Results

### Hypoxia induced the downregulation of PI3K and the upregulation in Caspase-3 in hAD-MSCs

**Fig. 1:**
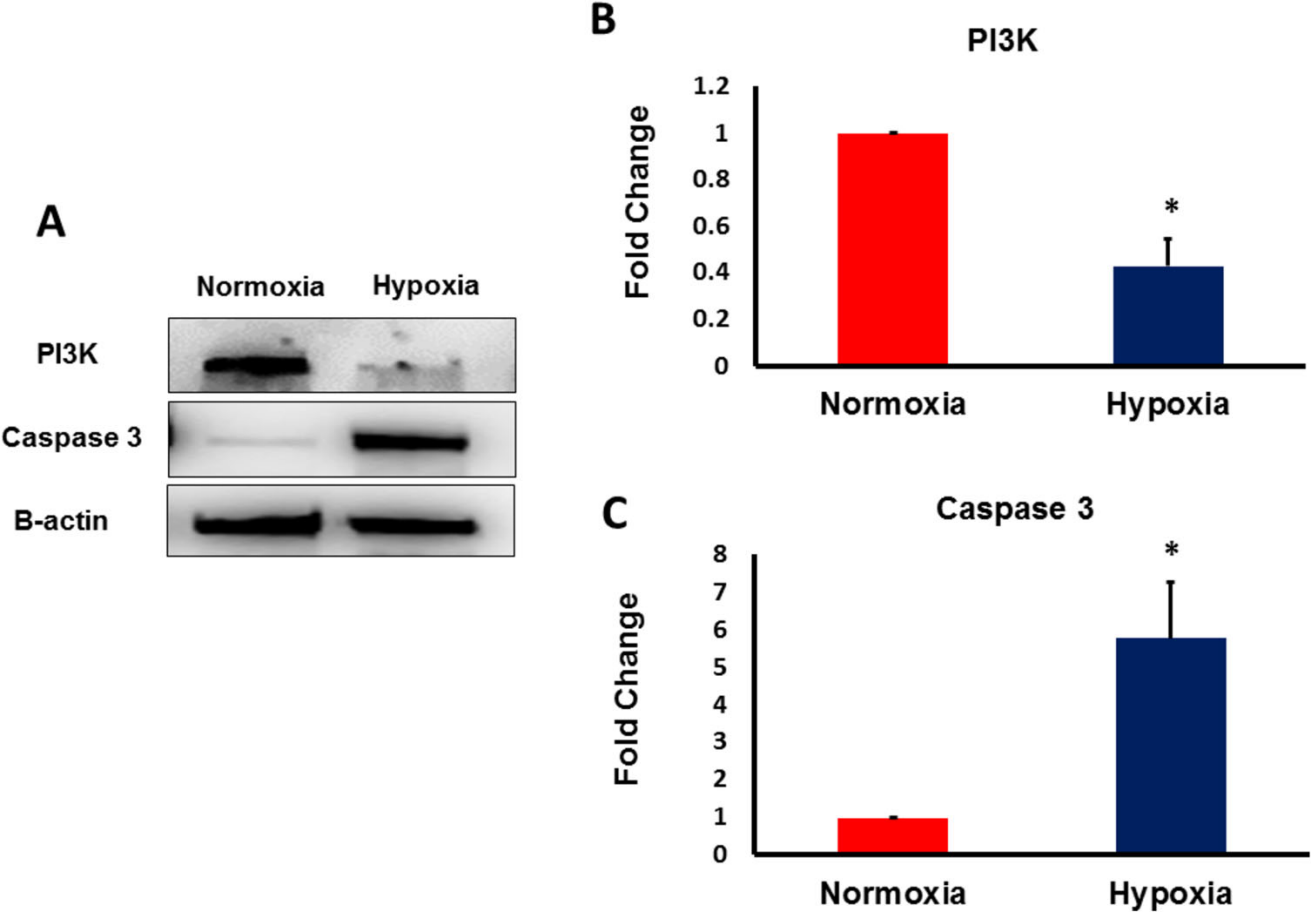
Exposure to severe hypoxia reduced the survival rate and increased the rate of cell death of MSCs. MSCs were cultured under hypoxia conditions induced with 400 μM CoCl_2_ for 48 h. **A** Representative Western blot analysis of PI3K and Caspase 3 expression in MSCs under severe hypoxia and normoxia. β actin was used for normalization of protein loading. **B** survival rate of MSCs under severe hypoxia was significantly lower than that of the control cells. **C** The rate of cell death in MSCs cultured under hypoxia was significantly increased compared to that of the control. (*p < 0.05 vs. control)

To confirm the induction of hypoxia following CoCl2 treatment, protein level of HIF-1α was detected using western blot and the results revealed significant induction of HIF-1α (supplementary Fig. 1A). The level of HIF-1α- associated inflammatory cytokine activation was also assessed. The level of tumor necrosis factor alpha (TNF-α) was remarkably upregulated in hypoxic hAD-MSCs (supplementary Fig. 1B). The survival of hAD-MSCs after being exposed to 400 µm CoCl2 was analyzed by detecting the protein level of phosphoinositide 3-kinase (PI3K). As shown in Fig. 1A, B, a pronounced downregulation in the protein level of PI3K was detected in hAD-MSCs that were exposed to hypoxia compared to normoxic control cells. We next examined the effect of hypoxia on the expression of pro-apoptotic marker; Caspase-3. As shown in Fig. 1A, C, there was a noticeable, upregulation in the expression of Caspase-3 in hAD-MSCs after being exposed to hypoxic stress compared to normoxic control cells. These data indicated that severe hypoxia disrupted the survival machinery in hAD-MSCs and increased their susceptibility to be eradicated by apoptosis.

### Hypoxia induced the downregulation of Factor H and CD59

**Fig. 2:**
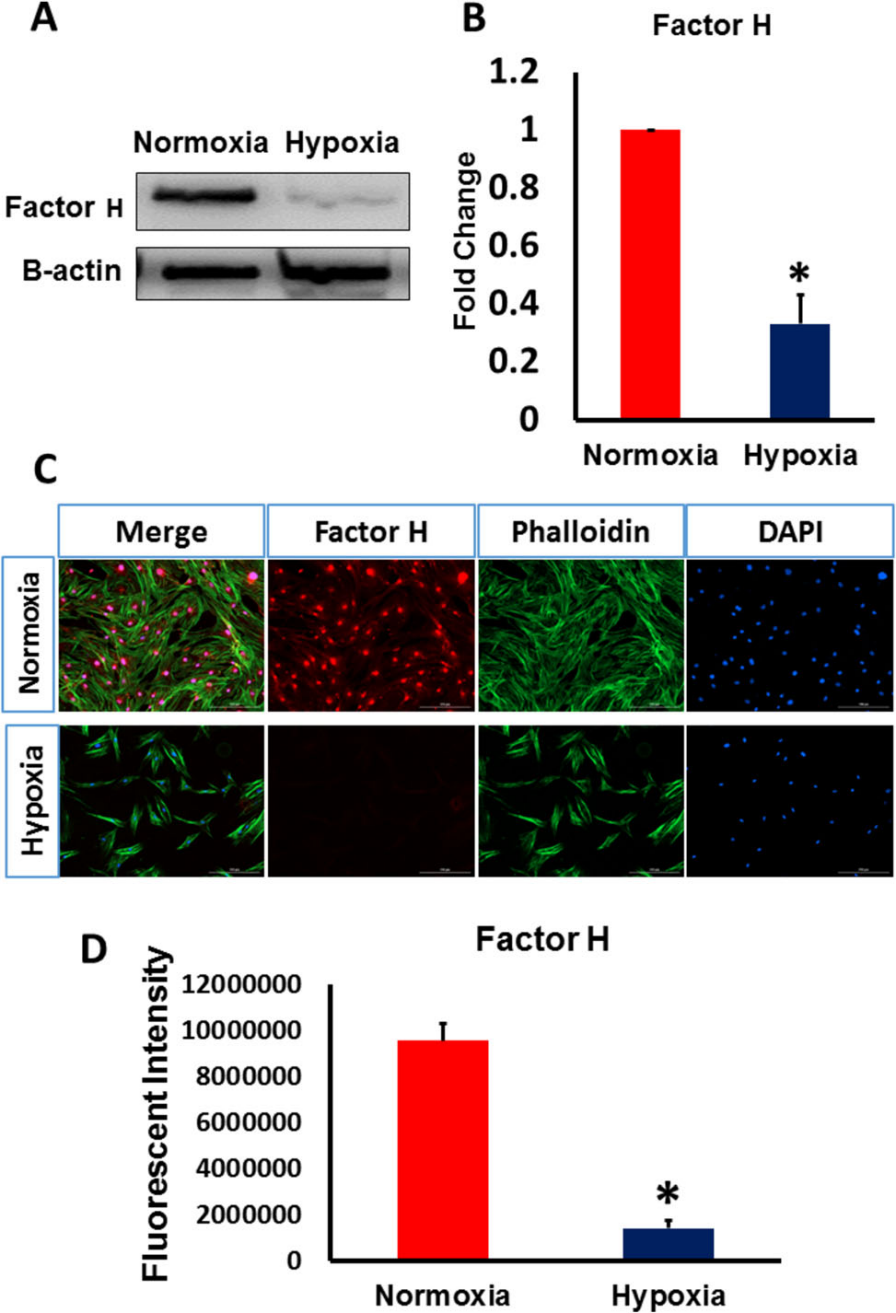
Exposure to severe hypoxia reduced Factor H production by MSCs. MSCs were exposed to hypoxia for 24 h, and Factor H levels were analyzed using Western blot and immunostaining. **A** Representative Western blot analysis of Factor H expression in MSCs under severe hypoxia and normoxia. β actin was used for normalization of protein loading. **B** Levels of Factor H measured by Western blotting were significantly lower in hypoxic MSCs than in MSCs exposed to normoxia. **C** Representative images of Factor H immunofluorescence in MSCs cultured under hypoxia and normoxia. **D** Immunofluorescence showed a significant decrease in the expression of Factor H under hypoxia compared to normoxia. (*p < 0.05 vs. control, n = 3)

**Fig. 3:**
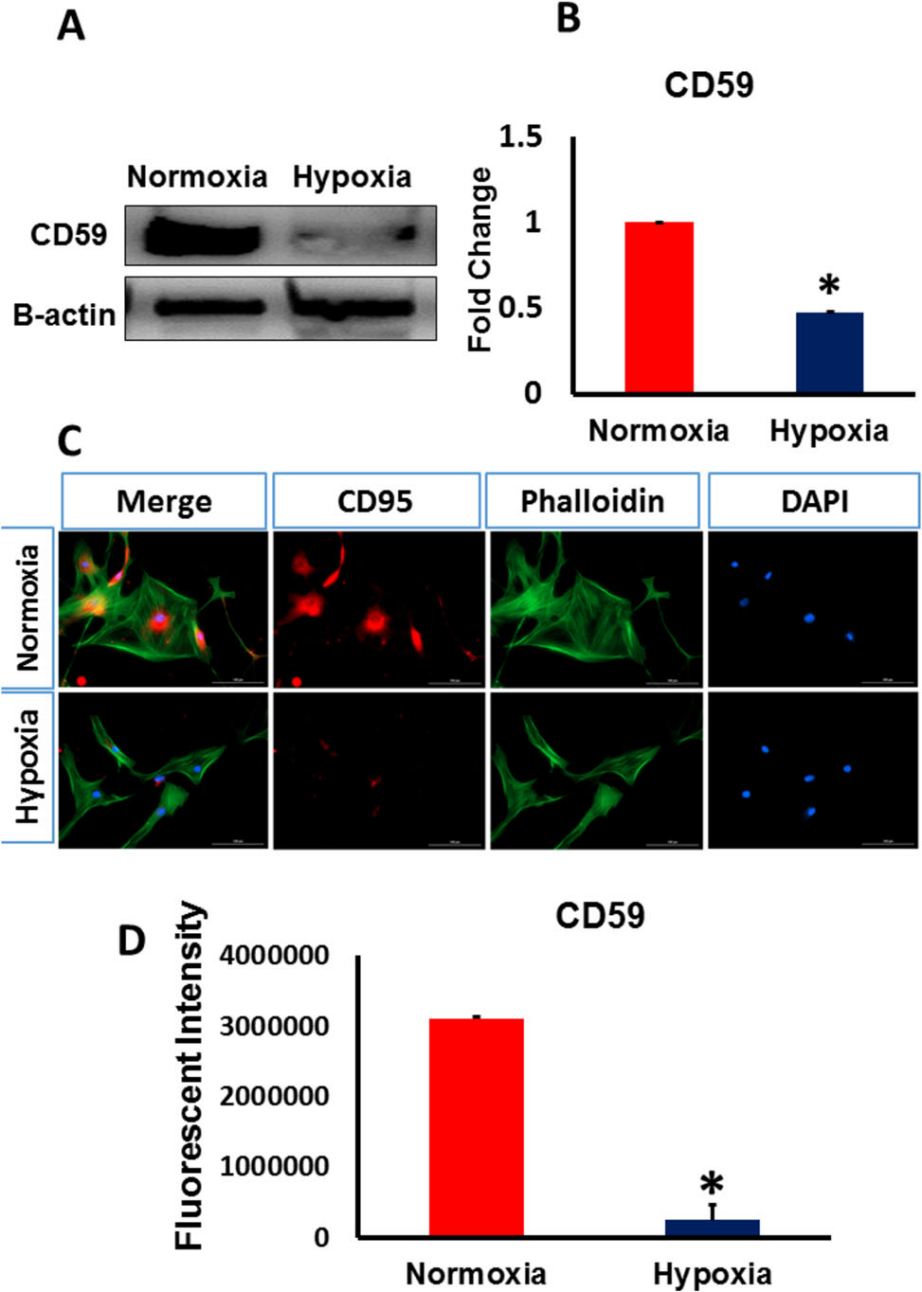
Exposure to severe hypoxia reduced the expression of CD59 in MSCs. MSCs were exposed to hypoxia for 24 h, and CD59 expression level was analyzed using Western blot and immunostaining. **A** Representative Western blot analysis of CD59 expression in MSCs under severe hypoxia and normoxia. β actin was used for normalization of protein loading. **B** CD59 expression levels as measured by Western blotting significantly decreased in hypoxic MSCs compared to normoxic cells. **C** Representative images of CD59 immunofluorescence in MSCs cultured under hypoxia and normoxia. **D** Immunofluorescence showed a significant decrease in the expression of CD59 under hypoxia compared to control. (*p < 0.05 vs. control, n = 3)

Factor H and CD59 are key regulators of the complement system by inhibiting the activation of C3 convertase and MAC complex, thus they are crucial for shutting down cell death pathways [17]. To investigate the effect of hypoxia on the protein level of Factor H and CD59, we used western blotting and immunocytochemistry to detect their levels. As depicted in Figs. 2A, B and 3A, B the protein level of Factor H and CD59 was significantly decreased in hypoxia –exposed hAD-MSCs compared to control cells. These western blotting results were further confirmed using immunocytochemistry which also revealed a remarkable decline in the expression of both Factor H and CD59 in hypoxic MSCs (Figs. 2C, D and 3C, D). These results indicated that severe hypoxia can negatively impact the expression of complement regulatory factors; Factor H and CD59, in MSCs and increase the possibility of inciting the complement cascade and its subsequent apoptotic pathway.

### The lack of Factor H and CD59 in hypoxic hAD-MSCs increased the formation and accumulation of C3b

**Fig. 4:**
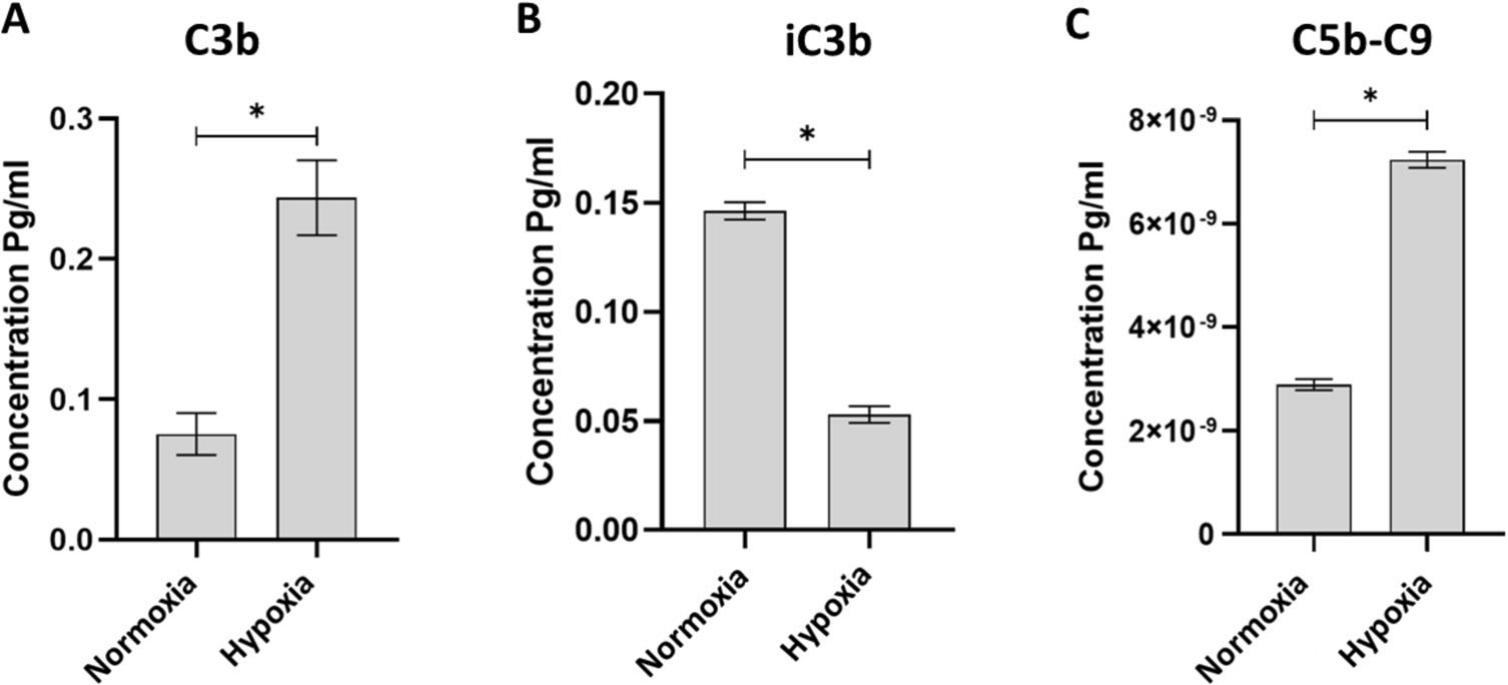
Exposure to severe hypoxia enhanced complement system activation within the MSCs. The expression level of the complement component markers C3b, iC3b and C5-C9 were analyzed using Elisa assay. By comparing the expression levels of the complement components C3b, iC3b, and C5-C9 in MSCs under hypoxic and normoxic conditions, the ELISA assay can identify dysregulation of complement activation in response to severe hypoxia. **A** C3b expression levels were significantly increased in hypoxic MSCs compared to control. **B** iC3b expression levels decreased significantly in hypoxic MSCs compared to normoxic cells. **D** C5-C9 expression levels were significantly higher in hypoxic MSCs compared to control. (*p < 0.05 vs. control, n = 3)

C3 is a central component where all three complement pathways converge. C3 is cleaved upon initiating the activation of complement cascade into C3a and the opsonin; C3b. If Factor H and CD59 are abundant, C3b will be hydrolyzed to form inactive C3b (iC3b), as a result the subsequent activation of complement factors will be terminated. To correlate the downregulation in the level of Factor H and CD59 in hypoxic MSCs with the incitement of complement cascade, we measured the activity and the expression of C3b and iC3b in normoxic and hypoxic MSCs using specific ELISA kits. The results showed that the absence of Factor H and CD59 in hypoxic MSCs was associated with high level of C3b and low level of iC3b compared to normoxic MSCs (Fig. 4A, B).

### The activation of C3b in hypoxic hAD-MSCs induced the formation and assembly of MAC complex

**Fig. 5:**
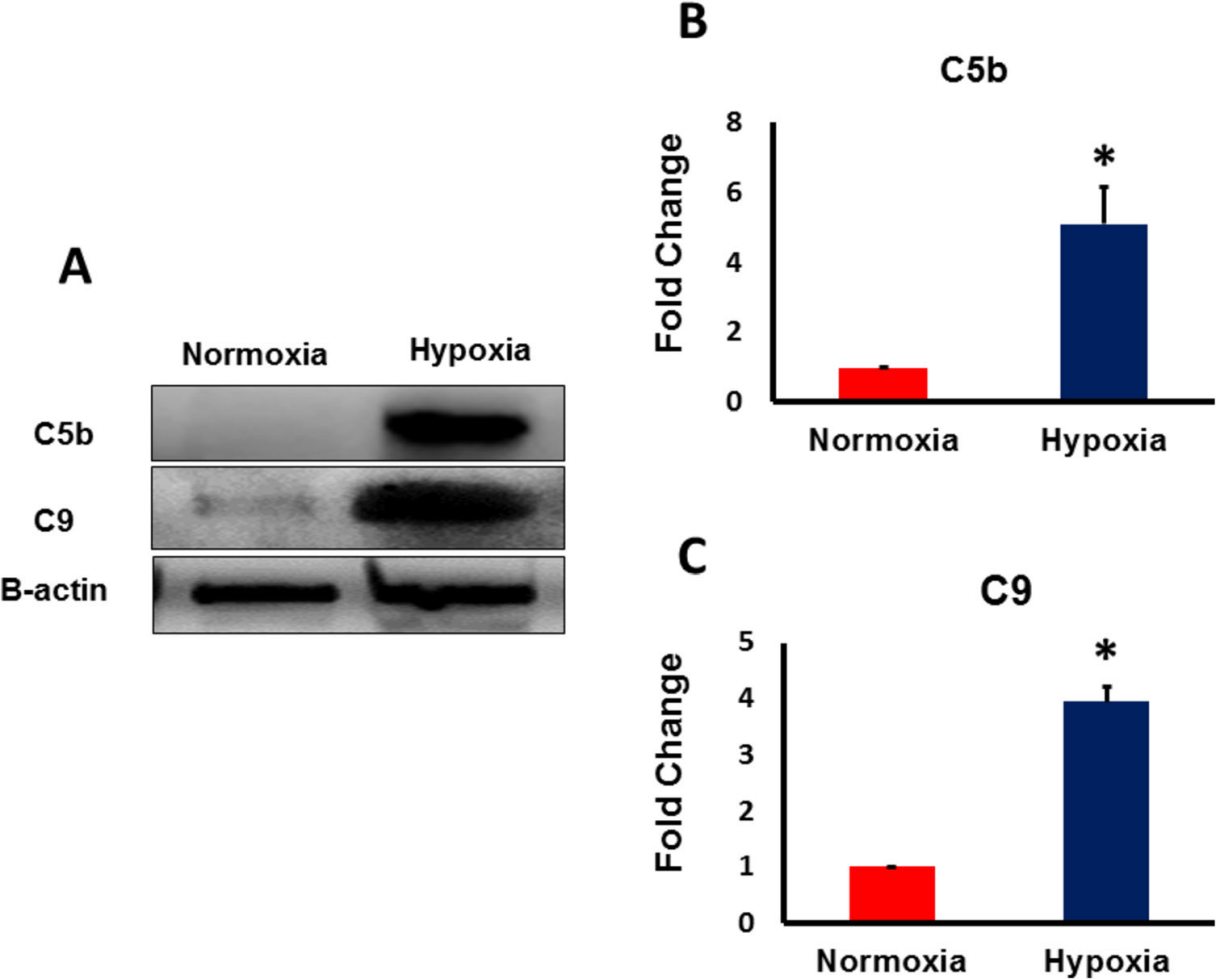
Molecular mechanisms of MAC assembly in severe hypoxia-exposed MSCs. MSCs were exposed to hypoxia for 24 h. **A** C5b and C9 levels were analyzed using Western blot. β actin was used for normalization of protein loading. The levels of both **B** C5b and **C** C9 increased significantly in hypoxic MSCs compared to MSCs under normoxia. (*p < 0.05 vs. control, n = 3)

MAC complex is the final product of activated complement system which is responsible of inducing cellular lysis and death. It is formed by the assembly of activated complement factors; C5b, C6, C7, C8, and C9. To confirm the formation of MAC complex following the activation of C3b in hypoxic MSCs, we used western blotting and MAC ELISA kit (C5b-C9 kit) to detect its level. As shown in Fig. 4C, MAC complex (C5b-C9) was formed and activated in hypoxic MSCs compared to the normoxic counterparts. Further, western blotting results depicted in Fig. 5A–C revealed significant upregulation in the protein level of two major components of MAC complex; C5b and C9, in hypoxic MSCs. The assembled and activated MAC complex can be inserted into the surface of hypoxic MSCs causing their lysis and damage. The formation of this destructive complex can explain the poor survival and high apoptotic rate of MSCs under hypoxic conditions.

## Discussion

MSCs-based therapy has shown promising results in preclinical and clinical studies for treating a wide range of diseases and conditions making them an ideal candidate for regenerative medicine. Despite exerting propitious therapeutic outcomes, many challenges are still there which hinder shifting them from bench to bedside. Several reports highlighted that MSCs exhibited poor survival and low retention rate after being transplanted at the host tissues [12]. These post-transplantation hurdles are more common in hypoxia-associated diseases, such as cardiovascular disorders [18]. MSCs favorably reside in hypoxic niches to support their stemness and proliferation as the level of hypoxia inside these niches is mild to moderate [19]. On the other hand, severe hypoxia is considered a harsh microenvironment for MSCs that can negatively impact their survival and regenerative abilities [20]. As severe hypoxia affects the therapeutic efficacy of MSCs, it is an imperative requisite to investigate its impact on MSCs biological and molecular characteristics. Understanding the molecular basis of hypoxia- mediated changes in MSCs may help in determining many factors that can be targeted genetically or pharmacologically to improve their survival in such a harsh microenvironment. Xing and co-authors reported in their study that chronic hypoxia existed in patients with cyanotic congenital heart disease induced premature senescence of Bone –marrow derived MSCs (BM-MSCs) [21]. Abu-El-Rub and co-authors revealed that severe hypoxia (0.4% O2) reduced the survival of BM-MSCs and increased their rejection rate [22].

No study so far has investigated the association between hypoxia and complement system modulation in MSCs. MSCs are known to suppress the complement cascade through producing Factor H and CD59. These factors prevent the activation of complement system and protect MSCs from being destroyed by MAC complex. Factor H and CD59 existing in MSCs are regarded as potent defensive mechanisms to promote their auto-protection from complement-mediated damage and increase their survival at the host site.

Referring to the importance of Factor H and CD59 in promoting the survival of MSCs at the site of infusion, it is worthwhile to investigate the correlation between severe hypoxia and these complement regulatory factors. Herein, we reported intriguing results that severe hypoxia can disturb the auto-protection mechanism in MSCs by downregulating Factor H and CD59. We can propose that hypoxic stress which is marked by the upregulation in the expression of HIF-1α, an important transcription factor for modulating many cellular signaling pathways, is responsible of decreasing the protein level of Factor H and CD59. The downregulation of these protective factors in hypoxic MSCs can trigger the assembly and formation of MAC complex causing their lysis and apoptosis. MAC complex initiated the lysis of cells by forming cytotoxic pores on their surface. Ziporen and co-authors found that MAC complex can trigger programmed necrotic cell death in mouse embryonic fibroblasts and primary fibroblasts mediated by activating caspases and BH3 interacting-domain death agonist (BID). Ng ESY and co-authors also reported that the activation of C3 complement factor and the insertion of MAC complex on the surface of retinal pigment epithelial (RPE) cells in patients suffering from Recessive Stargardt disease (STGD1) can increase their death rate and worsen the existing retinopathy. These studies supported our findings regarding the involvement of MAC complex in initiating the autolysis of MSCs independent of immune cells recruitment and activation. Preserving proper levels of Factor H and CD59 can promote the regenerative abilities of MSCs in hypoxia-associated pathologies. To conclude, the findings reported in our study provided new mechanistic explanation of the poor survival and high death rate of MSCs in severe hypoxic conditions. Furthermore, these findings can be utilized to develop effective strategies to improve the survival of MSCs in hypoxia-associated disorders. Genetic engineering of MSCs to overexpress Factor H or CD59 or both can be proposed as an effective strategy to increase their tolerance and survival in severe hypoxic conditions. Moreover, pharmacological or genetic inhibition of MAC complex can be suggested as another effective measure to keep MSCs protected and healthy in severe hypoxic microenvironments. The regulatory mechanism regarding the involvement of HIF-1α in modulating the expression of factor H and CD59 in hypoxic MSCs should be also investigated in future studies in order to improve the survival of MSCS in hypoxic conditions.

## Supplementary Information

Below is the link to the electronic supplementary material.

Supplementary file1 (DOCX 159 KB)

## Data Availability

The data that supports the findings in this study are available from the corresponding authors upon reasonable request.
