# Supplementary material for: Hypoxic Stress Induces Complement-Mediated Lysis of Mesenchymal Stem Cells by Downregulating Factor H and CD59
Source: Tissue Eng Regen Med. 2024 Nov 1;22(1):105–12. doi: 10.1007/s13770-024-00678-6 (PMC11711716; doi:10.1007/s13770-024-00678-6)
Supplement: Supplementary file 1 — Supplementary file1 (DOCX 159 KB) [file 13770_2024_678_MOESM1_ESM.docx]

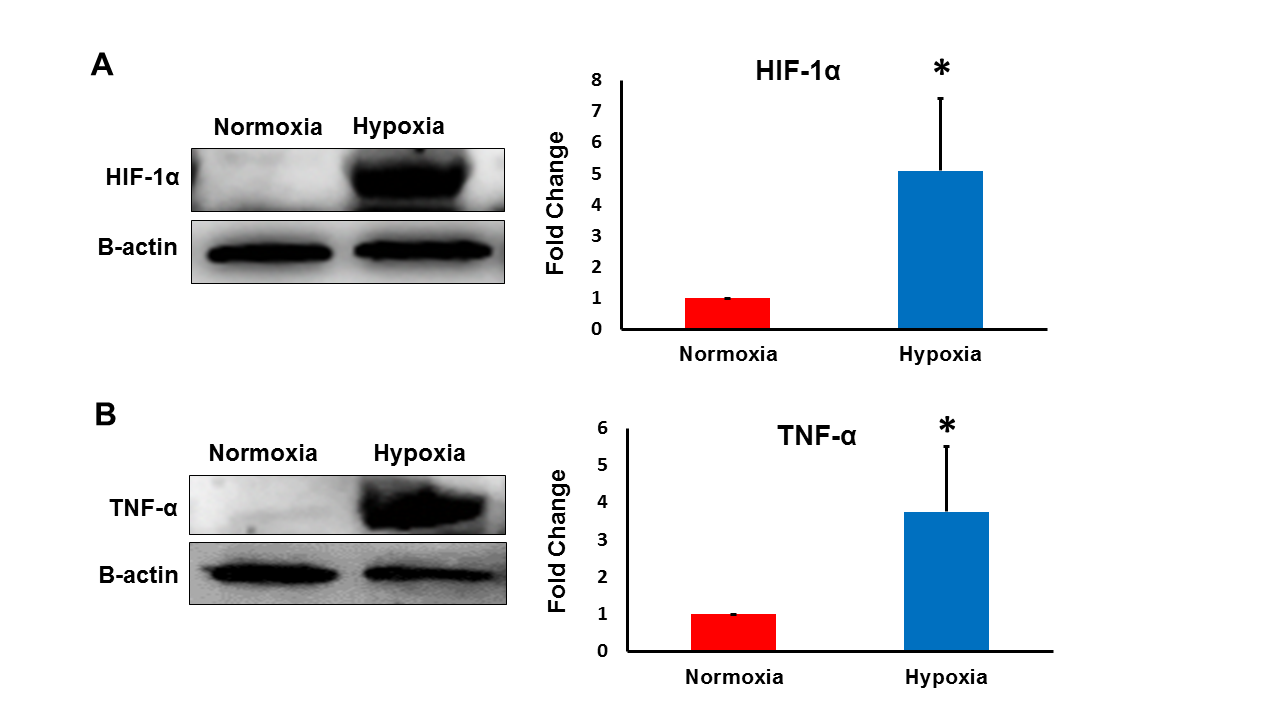


**Supplementary Figure 1: The protein expression of HIF-1α and TNF-α after hypoxia induction with CoCl2 in hAD-MSCs**. Western blotting analysis of A) **HIF-1α** and B) **TNF-α** showed remarkable upregulation in their level following the treatment of MSCs with COCl2. N=3, p<0.05 compared to normoxia (untreated MSCs).
